# Mitochondrial dynamics and their role in the pathogenesis of age-related macular degeneration: A comprehensive review

**DOI:** 10.1016/j.redox.2025.103976

**Published:** 2025-12-20

**Authors:** Kai-Yang Chen, Hoi-Chun Chan, Wan-Wan Lin, Chi-Ming Chan

**Affiliations:** a Department of General Medicine, Chang Gung Memorial Hospital (Linkou branch), Taoyuan, Taiwan; b School of Pharmacy, China Medical University, Taichung, Taiwan; c Department of Pharmacology, College of Medicine, National Taiwan University, Taipei, Taiwan; d Graduate Institute of Medical Sciences, Taipei Medical University, Taipei, Taiwan; e Department of Ophthalmology, Cardinal Tien Hospital, New Taipei City, Taiwan; f School of Medicine, Fu Jen Catholic University, New Taipei City, Taiwan

**Keywords:** Age-related macular degeneration, Mitochondrial dynamics, Retinal pigment epithelium, Mitochondrial dysfunction, Fission, Fusion, Biogenesis, Mitophagy

## Abstract

Age-related macular degeneration (AMD) is a leading cause of irreversible blindness in the elderly and has a multifactorial etiology involving advanced age, genetic susceptibility, and environmental risk factors. Accumulating evidence suggests that mitochondrial dysfunction is a central pathogenic mechanism in AMD, particularly in the retinal pigment epithelium (RPE). The RPE is critical for retinal homeostasis, and its high metabolic activity renders it vulnerable to age-related mitochondrial dysfunction. In AMD, the core processes of mitochondrial dynamics—fission, fusion, biogenesis, and mitophagy—are profoundly dysregulated, leading to a fragmented and dysfunctional mitochondrial network. This failure of quality control results in bioenergetic deficits, excessive oxidative stress, and the release of damage-associated molecular patterns that fuel chronic inflammation and complement-mediated damage. Experimental models and human tissue studies have strengthened the link between mitochondrial dysfunction and AMD pathology, revealing structural abnormalities, mitochondrial DNA (mtDNA) damage, and altered metabolic signatures. Therapeutic strategies targeting mitochondrial pathways, including mitochondria-targeted antioxidants, dynamic modulators, and enhancers of biogenesis and mitophagy, such as agents that restore defective mitophagosome formation, represent promising avenues for intervention. As the field advances, the integration of biomarker development and personalized approaches holds the potential to transform the clinical landscape of AMD by addressing the root causes of cellular dysfunction.

## 1 Introduction and background

Age-related macular degeneration (AMD) is the leading cause of irreversible blindness in elderly populations worldwide, presenting a substantial challenge to public health systems and severely impairing the quality of life of affected individuals, who often experience depression, social withdrawal and increased economic dependency [[1], [2], [3]]. The global burden of AMD is projected to escalate, with estimates suggesting that it will affect approximately 288 million people by 2040, a trend fueled by increasing life expectancy and lifestyle factors [1]. Clinically, AMD is classified into two primary forms: the more prevalent dry (atrophic) AMD, characterized by drusen deposition and geographic atrophy, and the neovascular (wet) AMD, defined by choroidal neovascularization and exudation, which often leads to more rapid and severe vision loss [4,5].

The etiology of AMD is multifactorial, involving an interplay of advanced age, genetic susceptibility (particularly in genes related to the complement pathway and lipid metabolism), and environmental risk factors, such as smoking [5,6]. While therapeutic advances, notably anti-vascular endothelial growth factor (VEGF) agents, have improved outcomes for neovascular AMD, no effective treatment exists for the geographic atrophy that defines advanced dry AMD, highlighting the urgent need to elucidate its underlying pathogenic mechanisms [6].

The retinal pigment epithelium (RPE) is central to the pathophysiology of AMD. This monolayer of highly specialized cells lies at the critical interface between the neural retina and the choroid. Its functions are vital for retinal homeostasis, including the phagocytosis of shed photoreceptor outer segments, recycling of visual pigments in the retinoid cycle, metabolite transport, and regulation of immune privilege [7]. The high metabolic activity of the RPE and its perpetual exposure to light and oxidative stress render it particularly vulnerable to cumulative damage, with mitochondrial dysfunction emerging as a key component of its age-related decline and a central driver of AMD pathology [7,8].

Mitochondria are dynamic organelles that undergo continuous remodelling through coordinated fission, fusion, biogenesis, and mitophagy. These dynamics are essential for maintaining a healthy mitochondrial network by regulating morphology, distributing mitochondria, replenishing the mitochondrial pool, and eliminating damaged units [9]. Fission, mediated by Drp1, facilitates mitochondrial division and the isolation of dysfunctional segments. Fusion, governed by mitofusins (Mfn1/2) and OPA1, allows for the complementation of mitochondrial content. Biogenesis, driven by transcriptional regulators such as PGC-1α, generates new mitochondria. Mitophagy, the selective autophagy of mitochondria via the PINK1/Parkin pathway, is a critical quality-control mechanism [9]. This process proceeds through the ordered initiation, elongation, and sealing of a double-membraned mitophagosome that specifically engulfs defective mitochondria. In the retina, which has one of the highest energy demands in the body, a precise balance between these processes is indispensable for cellular health. Dysregulation can lead to bioenergetic failure, excessive oxidative stress, and ultimately, the death of RPE and photoreceptor cells, underpinning the pathogenesis of AMD [8,10].

## 2 Retinal structure and function

The retina is a complex, layered neural tissue whose functional integrity is critically dependent on the RPE. This single layer of polygonal cells forms the outer blood-retina barrier, tightly regulating the movement of nutrients, ions, and waste between the photoreceptors and the choroidal blood supply, while also providing a barrier against neovascular infiltration. The dark appearance of the RPE, due to its high melanin concentration, serves to absorb stray light, minimizing phototoxic scatter. Furthermore, it is equipped with robust metabolic and antioxidant systems to manage the high reactive oxygen species (ROS) load generated by the intense metabolic activity and light exposure of the retina [7,11].

### 2.1 Key functions of the RPE

The RPE sustains retinal health through several indispensable functions.

• **Phagocytosis of Photoreceptor Outer Segments:** Daily phagocytosis and degradation of shed photoreceptor outer segments are essential for photoreceptor renewal and prevention of toxic debris accumulation. This process is mediated by specific receptors, including MerTK and αvβ5 integrin [12].
• **Visual Cycle Regulation:** The RPE is integral to the retinoid (visual) cycle, regenerating 11-*cis* retinal from all-*trans* retinal for reuse in phototransduction. Mutations in genes critical to this process, such as RPE65, cause severe retinal dystrophies, underscoring its importance [13].
• **Barrier Function and Immune Regulation:** As part of the outer blood-retina barrier, the RPE controls immune cell access and secretes cytokines that help maintain retinal immune homeostasis, a function increasingly implicated in AMD [7,11].

### 2.2 Photoreceptor energetics and mitochondrial specialization

Photoreceptors are neurons with exceptional metabolic demands, requiring vast amounts of adenosine triphosphate (ATP) for phototransduction and the constant renewal of their outer segments. This makes them heavily reliant on mitochondrial oxidative phosphorylation [14]. Beyond their role as powerhouses, mitochondria in the retina exhibit specialized functions; for example, their specific arrangement in photoreceptor inner segments has been proposed to act as a microlens, funnelling light efficiently to the outer segments and thereby optimizing phototransduction [15].

### 2.3 Mitochondrial roles in retinal physiology

Mitochondria are crucial for retinal health through their roles in:

• **ATP Production:** Fuelling the energy-intensive processes of vision.
• **Oxidative Stress Management:** Generating and simultaneously buffering ROS to maintain redox balance.
• **Calcium Signaling and Metabolite Processing:** Regulating intracellular calcium for signaling and processing vital metabolites like iron and cholesterol.

Disruption of these mitochondrial processes—through DNA damage, impaired dynamics, or inadequate quality control—directly compromises RPE and photoreceptor viability, establishing mitochondrial dysfunction as a cornerstone of AMD pathogenesis [8,10].

## 3 Mitochondrial dynamics: mechanisms and molecular regulation

The functional integrity of the mitochondrial network is governed by a precise equilibrium between opposing processes: fission and fusion, balanced by biogenesis and mitophagy. This dynamic remodelling is orchestrated by a suite of highly conserved GTPase proteins as summarized in Table 1 [9,16].

**Table 1:** Core molecular machinery of mitochondrial dynamics.

Process	Key Proteins	Primary Function	Implications in AMD Pathogenesis	References
**Fission**	Drp1, Fis1, MFF	Divides mitochondria; isolates damaged segments; facilitates apoptosis.	Excessive fission leads to mitochondrial fragmentation, bioenergetic inefficiency, and increased ROS production.	[16,17]
**Fusion**	Mfn1, Mfn2, OPA1	Merges mitochondria; mixes contents; compensates for defects.	Reduced fusion impairs complementation, allowing defective mitochondria to accumulate.	[[18], [19], [20]]
**Mitophagy**	PINK1, Parkin	Selectively tags and removes damaged mitochondria via autophagy.	Impaired clearance leads to accumulation of dysfunctional, ROS-producing organelles.	[[21], [22], [23]]
**Biogenesis**	PGC-1α, NRF-1/2, TFAM	Generates new mitochondria to meet energy demand and maintain the network.	Downregulation results in a dwindling pool of functional mitochondria, leading to energy crisis.	[22,24]

**Fig. 1:**
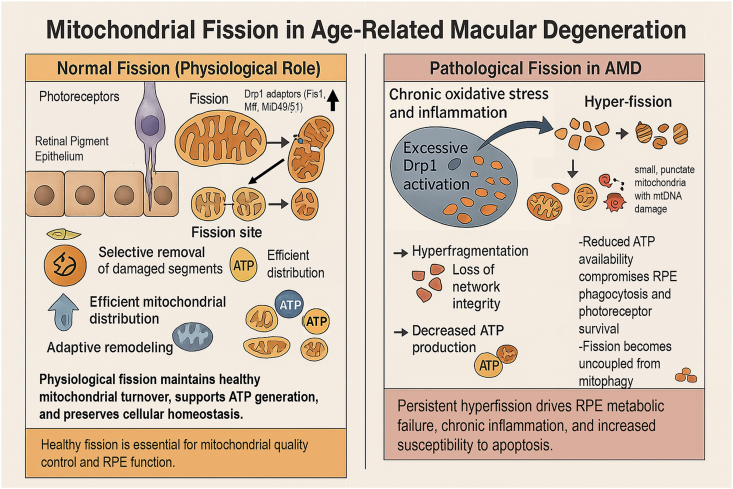
Physiological versus pathological mitochondrial fission in the retina in AMD. Left panel: In photoreceptors and the retinal pigment epithelium (RPE), localized activation of the fission GTPase dynamin-related protein 1 (Drp1) at defined fission sites, coordinated by the outer-membrane adaptor proteins Fis1, mitochondrial fission factor (MFF) and mitochondrial dynamics proteins of 49 and 51 kDa (MiD49/51), permits selective removal of damaged mitochondrial segments, efficient distribution of ATP-generating organelles, and adaptive remodelling of the network, thereby maintaining mitochondrial turnover and cellular homeostasis. Right panel: In AMD, chronic oxidative stress and inflammation drive excessive Drp1 activation and mitochondrial hyper-fission, generating a highly fragmented network of small, punctate mitochondria with mitochondrial DNA (mtDNA) damage, loss of network integrity, reduced ATP production and fission events that are uncoupled from mitophagy, which together impair RPE phagocytosis and photoreceptor survival and promote RPE metabolic failure, chronic inflammation and increased susceptibility to apoptosis.

Fission facilitates the division of mitochondria, a process critical for their distribution during cell division, the isolation of damaged segments, and the initiation of apoptosis. The central regulator of fission is dynamin-related protein 1 (Drp1), which is recruited from the cytosol to the outer mitochondrial membrane (OMM) by adaptor proteins such as mitochondrial fission 1 protein (Fis1), mitochondrial fission factor (MFF), and MiD49/51. Once assembled, Drp1 oligomerizes and constricts the mitochondrion, culminating in membrane scission [16,17]. The physiological and pathological roles of Drp1-mediated fission in the retina are summarized in Fig. 1. Under basal conditions in photoreceptors and the RPE, localized Drp1 activation at defined fission sites, coordinated by adaptor proteins Fis1, MFF and MiD49/51, enables selective removal of damaged mitochondrial segments, efficient distribution of mitochondria to high-demand regions, and adaptive remodelling of the network, thereby sustaining ATP production and cellular homeostasis. In AMD, chronic oxidative and inflammatory stress drive excessive Drp1 activation and mitochondrial hyperfission, resulting in a highly fragmented network of small, punctate mitochondria with mitochondrial DNA (mtDNA) damage, reduced ATP output, and fission events that become uncoupled from mitophagy. This pathological shift compromises RPE phagocytosis and photoreceptor survival and sets the stage for the downstream degenerative cascade described in Section 4.3.

**Fig. 2:**
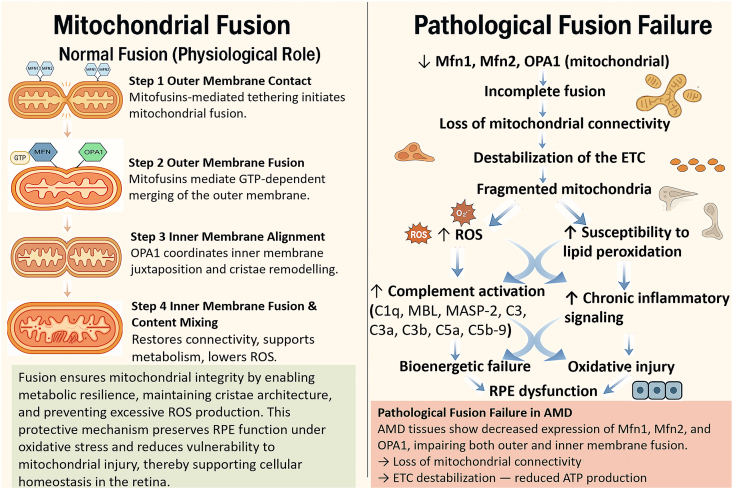
Normal mitochondrial fusion and pathological fusion failure in AMD. Left panel: Physiological mitochondrial fusion proceeds through a sequential four-step process in which mitofusins Mfn1 and Mfn2 mediate outer membrane contact and GTP-dependent outer membrane fusion, followed by OPA1-driven inner membrane alignment, cristae remodelling, and inner membrane fusion with matrix content mixing. This ordered sequence restores network connectivity, maintains cristae architecture, supports efficient oxidative metabolism, and limits excessive reactive oxygen species (ROS) production, thereby preserving retinal pigment epithelium (RPE) function. Right panel: In AMD, decreased expression of Mfn1, Mfn2 and OPA1 leads to incomplete fusion, loss of mitochondrial connectivity, destabilization of the electron transport chain (ETC), and accumulation of fragmented mitochondria that generate excess ROS, heightening susceptibility to lipid peroxidation. The resulting oxidative stress promotes complement activation (mannose-binding lectin (MBL), MBL-associated serine protease 2 (MASP-2), C1q, C3, C3a, C3b, C5a, and C5b-9 membrane attack complex), chronic inflammatory signaling, bioenergetic failure and RPE dysfunction.

Fusion counteracts fission by merging the contents of adjacent mitochondria, promoting the homogenization of metabolites, proteins, and mtDNA, thereby rescuing partially damaged units. This protective process is mediated by three key GTPases and occurs through a coordinated, sequential four-step mechanism illustrated in Fig. 2: OMM contact is first achieved through the interaction of the mitofusins (Mfn1 and Mfn2) on adjacent mitochondria, which is then immediately followed by the OMM fusion (Outer Mitochondrial Membrane fusion) executed by these same proteins [18]. Subsequently, IMM contact takes place between the inner membranes, which in turn leads to IMM fusion (Inner Mitochondrial Membrane fusion) executed by optic atrophy 1 (OPA1), thereby merging the matrix contents. Under normal conditions, this ordered sequence—outer-membrane merging first, followed by inner-membrane merging—is coordinated by Mfn1/Mfn2 and OPA1, respectively. This preserves cristae structure, maintains metabolic resilience, enables mitochondrial content complementation, and limits excessive ROS formation, thereby supporting cellular homeostasis in the retina. Conversely, AMD tissues exhibit downregulation of Mfn1, Mfn2, and OPA1, leading to incomplete fusion, loss of mitochondrial connectivity, and destabilization of the electron transport chain (ETC). The resulting fragmented mitochondria generate increased ROS and are highly susceptible to lipid peroxidation, which in turn amplifies complement activation and chronic inflammatory signaling within the RPE. Together, these abnormalities contribute to bioenergetic decline, oxidative injury, and progressive RPE dysfunction.

**Fig. 3:**
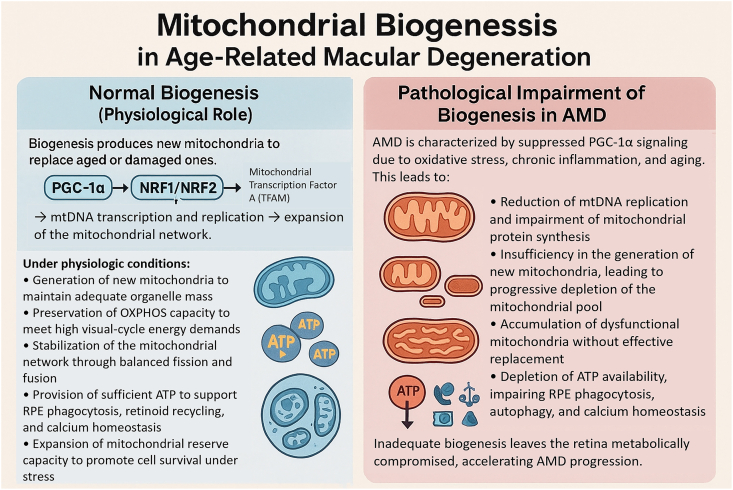
Mitochondrial biogenesis in the RPE under physiological conditions and in AMD. Left panel: In healthy RPE, activation of PGC-1α co-activates NRF1/NRF2 and upregulates TFAM, promoting mtDNA transcription and replication and expansion of the mitochondrial network. Adequate biogenesis maintains organelle mass, preserves oxidative phosphorylation capacity for high visual-cycle energy demands, and, together with fission and fusion, sustains a dynamic but stable mitochondrial network and mitochondrial reserve capacity. Right panel: In AMD, chronic oxidative stress, inflammation, and aging suppress PGC-1α signaling, leading to reduced mtDNA replication and defective synthesis of mitochondrial proteins. Biogenesis becomes insufficient to replace damaged organelles, resulting in depletion of the functional mitochondrial pool, accumulation of dysfunctional mitochondria, and decreased ATP production. This energetic deficit impairs RPE phagocytosis, autophagy, and calcium handling, leaving the retina metabolically compromised and promoting AMD progression.

Biogenesis expands the mitochondrial network in response to increased energy demand and is primarily regulated by the transcriptional coactivator PGC-1α (peroxisome proliferator-activated receptor-gamma coactivator 1-alpha). PGC-1α activates nuclear respiratory factors (NRF-1 and NRF-2), which in turn promote the expression of nuclear-encoded mitochondrial genes and the master regulator of mtDNA transcription and replication, TFAM (mitochondrial transcription factor A) (Fig. 3) [24]. As illustrated in Fig. 3, under physiological conditions in the RPE, the PGC-1α–NRF1/NRF2–TFAM axis drives mtDNA transcription and replication, thereby maintaining adequate mitochondrial mass, oxidative phosphorylation (OXPHOS) capacity, and a dynamic yet stable network. This ensures sufficient ATP generation to support high visual-cycle energy demands and ATP-dependent processes such as phagocytosis, retinoid recycling, and calcium homeostasis. In AMD, chronic oxidative and inflammatory stress suppresses this biogenic program, resulting in reduced mtDNA replication and impaired synthesis of respiratory chain proteins. Consequently, the mitochondrial pool becomes progressively depleted, dysfunctional mitochondria accumulate, and ATP availability declines, compromising RPE functions and accelerating tissue degeneration.

**Fig. 4:**
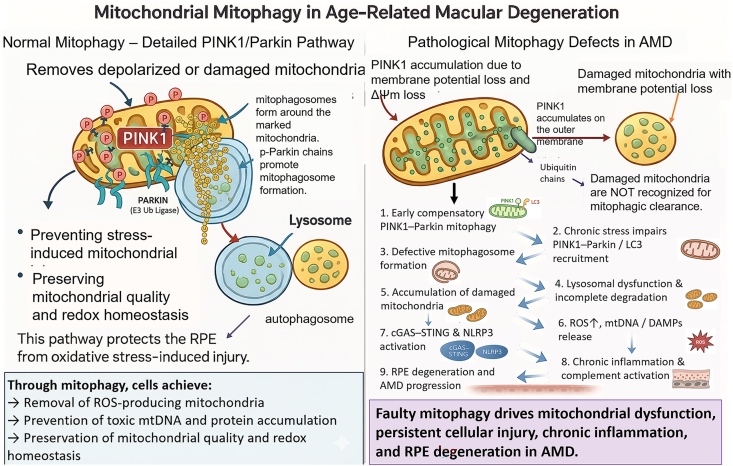
Physiological PINK1–Parkin–mediated mitophagy and its pathological failure in AMD. Left panel: Under normal conditions, loss of mitochondrial membrane potential (ΔΨm) stabilizes PINK1 on the outer mitochondrial membrane, leading to phosphorylation of ubiquitin and recruitment and activation of the E3 ubiquitin ligase Parkin, which ubiquitinates outer-membrane proteins to promote selective encapsulation of damaged mitochondria by the mitophagosome and subsequent lysosomal degradation, thereby preventing accumulation of ROS-producing organelles and preserving mitochondrial redox homeostasis in the RPE. Right panel: In AMD, although early compensatory PINK1 accumulation may occur, chronic oxidative and inflammatory stress impairs Parkin and microtubule-associated protein 1 light chain 3 (LC3) recruitment, disrupts mitophagosome formation, and compromises lysosomal degradation, resulting in accumulation of dysfunctional mitochondria with persistent ROS production and release of mitochondrial DNA (mtDNA) and other damage-associated molecular patterns (DAMPs), which activate cyclic GMP–AMP synthase (cGAS)–stimulator of interferon genes (STING) signaling and the NLR family pyrin domain-containing 3 (NLRP3) inflammasome, drive chronic inflammation and complement activation, and culminate in progressive RPE degeneration and AMD progression.

Mitophagy, the selective autophagic removal of damaged mitochondria, is a fundamental mitochondrial quality-control mechanism, principally regulated by the PINK1–Parkin pathway. Under physiological conditions, PINK1 is continuously imported into healthy mitochondria and rapidly degraded. Upon mitochondrial depolarization, however, PINK1 accumulates on the outer mitochondrial membrane (OMM), where it phosphorylates ubiquitin and recruits the E3 ubiquitin ligase Parkin. Activated Parkin ubiquitinates multiple OMM proteins, thereby labeling the damaged organelle for sequestration into a mitophagosome (mitochondria-containing autophagosome) and subsequent lysosomal degradation (Fig. 4) [21]. Through this tightly controlled process, the selective clearance of dysfunctional mitochondria prevents excessive ROS accumulation and preserves mitochondrial homeostasis. In AMD, this protective pathway becomes progressively compromised; despite PINK1 accumulation on impaired mitochondria, insufficient Parkin recruitment together with declining lysosomal capacity results in defective mitochondrial clearance. Consequently, the persistence of damaged mitochondria sustains pathological ROS production and activates innate immune signaling cascades, including TLR9 and cGAS–STING, thereby amplifying chronic inflammation and driving RPE degeneration.

The precise coordination of these processes is essential for adapting to metabolic stress and maintaining cellular health, with their dysregulation being particularly detrimental in post-mitotic, high-energy tissues like the retina.

## 4 Pathogenesis of AMD: the mitochondrial perspective

**Fig. 5:**
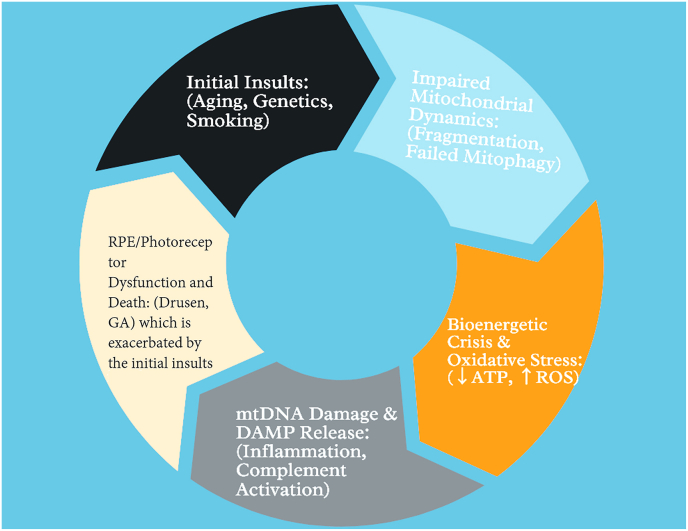
The self-perpetuating vicious cycle of mitochondrial dysfunction in age-related macular degeneration. In RPE cells, impaired mitochondrial dynamics characterized by excessive fission and reduced fusion leads to OXPHOS dysfunction, resulting in bioenergetic failure and increased production of ROS. Excess ROS drives oxidative stress, induces mtDNA damage, and promotes cytochrome *c* release, further exacerbating mitochondrial injury. Concurrently, defective mitophagy and impaired mitochondrial quality-control pathways lead to inefficient mitophagosome–lysosome processing and accumulation of dysfunctional mitochondria. The progressive buildup of damaged organelles amplifies oxidative stress and cellular injury, contributing to drusen formation at Bruch's membrane and loss of metabolic support to overlying rods and cones. These interlinked processes reinforce a continuous pathogenic feedback loop that drives RPE degeneration and photoreceptor loss in AMD.

Accumulating evidence positions mitochondrial dysfunction as a central and early pathogenic mechanism in AMD, potentially acting as a key driver of disease progression rather than merely a late-stage consequence [8,10]. The convergence of genetic susceptibility, environmental stressors, and the aging process creates a perfect storm of mitochondrial impairment within the RPE and photoreceptors. While the precise temporal sequence of events—whether mitochondrial damage precedes or results from other insults like complement activation—remains an active area of investigation, the interplay between these pathways creates a self-perpetuating vicious cycle [25,26]. In this cycle, mitochondrial damage leads to bioenergetic failure and oxidative stress, which in turn fuels further mitochondrial and cellular damage, relentlessly driving RPE and photoreceptor degeneration. This sequence of mitochondrial injury, bioenergetic failure, mtDNA destabilization, inflammatory signaling, and complement activation corresponds directly to the pathogenic loop illustrated in Fig. 5. The pathogenic loop can be conceptualized as a continuous vicious cycle centred on mitochondrial dysfunction in the RPE and its downstream impact on the neurosensory retina. Initial mitochondrial injury in RPE cells arises from aging, cumulative photo-oxidative stress, cigarette smoke exposure, and the exceptionally high metabolic demand of the macula, which together induce mtDNA damage, impair ETC efficiency, dissipate the mitochondrial membrane potential (ΔΨm), and diminish ATP production. As respiratory chain function declines, damaged mitochondria leak electrons to molecular oxygen and generate excessive ROS. These ROS species drive lipid peroxidation, protein oxidation, and further mtDNA injury, thereby amplifying mitochondrial dysfunction through a feed-forward mechanism that progressively destabilizes the organellar network.

In parallel, mitochondrial quality control becomes increasingly compromised. PINK1–Parkin–mediated mitophagy, which under physiological conditions selectively identifies and removes depolarized mitochondria, gradually loses efficiency. Defects in PINK1 stabilization, Parkin recruitment, mitophagosome biogenesis, and lysosomal degradation lead to failed clearance of damaged organelles. Autophagosome formation and lysosomal processing are impaired, causing dysfunctional mitochondria to accumulate within RPE cells. These damaged mitochondria, together with destabilized mitophagosomes, release a range of mitochondrial damage-associated molecular patterns (DAMPs), including oxidized mtDNA, cardiolipin, and oxidized mitochondrial peptides, into the cytosol and extracellular space, where they are sensed as potent “danger” signals by the innate immune system.

The liberation of mitochondrial DAMPs initiates and sustains inflammatory signaling cascades. Cytosolic and extracellular mtDNA activates the cGAS–STING pathway, while mitochondrial ROS and mtDAMPs promote assembly and activation of the NLRP3 inflammasome, culminating in the maturation and secretion of pro-inflammatory cytokines such as interleukin-1β (IL-1β) and interleukin-18 (IL-18). This innate immune activation converges with chronic oxidative injury to drive sustained complement system overactivation, particularly of the alternative pathway. Complement components including C3 and the terminal C5b-9 membrane attack complex (MAC) become persistently engaged on the surface of RPE and choroidal cells. Sublytic and lytic MAC deposition disrupts RPE cell membranes, triggers calcium influx, and further perturbs mitochondrial function, thereby reinforcing organellar stress. As a consequence, RPE cells progressively lose their capacity to phagocytose photoreceptor outer segments, to support the visual cycle and retinoid transport, and to maintain tight metabolic coupling with overlying photoreceptors. Photoreceptors, deprived of trophic and metabolic support, experience profound energy insufficiency, heightened mitochondrial stress, and activation of intrinsic apoptotic pathways, leading to progressive cell death. The resulting degeneration of both RPE and photoreceptors intensifies oxidative stress and mtDAMP release, locking the retina into a self-propagating, irreversible positive feedback loop of mitochondrial damage, inflammation, complement activation, and neuronal loss that characterizes advanced AMD. Together, these interconnected events establish a self-reinforcing pathogenic circuit linking mitochondrial failure, inflammation, complement dysregulation, and retinal cell loss, as schematically summarized in Fig. 5.

### 4.1 The nexus of risk factors and mitochondrial insult

Aging, the foremost risk factor for AMD, is associated with a progressive decline in mitochondrial function, including decreased respiratory capacity and increased ROS production. This is compounded by genetic variants in genes such as complement factor H (CFH) and the ARMS2/HTRA1 locus (Age-Related Maculopathy Susceptibility 2 and High-Temperature Requirement A1). For CFH, impaired regulation of the complement cascade leads to chronic low-grade inflammation and sublytic attack on the RPE, which can secondarily induce mitochondrial dysfunction and oxidative stress [27]. The mechanism linking the *ARMS2*/*HTRA1* locus to mitochondrial health is less clear but may involve increased susceptibility to oxidative stress and impaired cellular stress responses [28]. Environmental stressors, particularly smoking, introduce a high oxidant load that directly damages mitochondrial components, overwhelming endogenous antioxidant defences and accelerating cellular senescence in the retina [29].

### 4.2 Oxidative stress, mtDNA damage, and failed quality control

The retina's high oxygen consumption and light exposure make it a hotspot for oxidative stress. Mitochondria are both a primary source and a key target of ROS, leading to oxidation of lipids, proteins, and mtDNA. In experimental AMD-like models, sodium iodate and methylglyoxal induce marked mitochondrial dysfunction, excessive fission, and autophagy/mitophagy activation accompanied by accelerated mitophagosome production in RPE cells, highlighting how metabolic stress directly reprograms mitochondrial dynamics and quality control [[30], [31], [32]]. The accumulation of mtDNA deletions and mutations in RPE cells from AMD donors has been directly documented, leading to encoded respiratory chain defects and a vicious cycle of further ROS generation [33].

### 4.3 Disrupted dynamics in AMD pathology

**Fig. 6:**
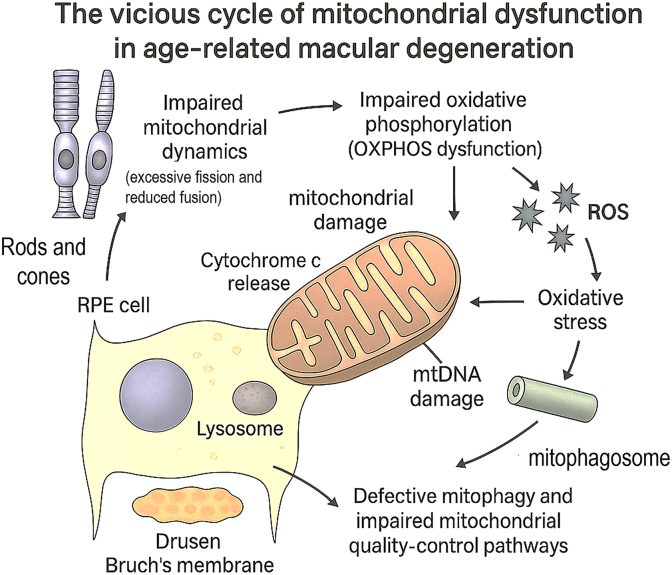
Integrated pathogenic loop linking mitochondrial dysfunction to retinal degeneration in AMD. Initial insults including aging, genetic susceptibility, and smoking initiate impairment of mitochondrial dynamics characterized by fragmentation and failed mitophagy, which precipitates a bioenergetic crisis with reduced ATP production and increased ROS generation. The resulting oxidative stress induces mtDNA damage and promotes the release of mitochondrial DAMPs, triggering chronic inflammation and complement activation. These processes converge to drive progressive RPE and photoreceptor dysfunction and cell death, manifested clinically by drusen formation and GA, which in turn exacerbate mitochondrial stress and reinforce this self-sustaining pathogenic cycle.

A shift in the balance of mitochondrial dynamics towards excessive fission is a hallmark of AMD models. Increased Drp1 activity and reduced Mfn2 expression lead to mitochondrial fragmentation, which compromises energy production and sensitizes cells to apoptosis [34,35]. These changes are accompanied by impaired Mfn1/Mfn2-and OPA1-dependent fusion, depicted in the pathological panel of Fig. 2, resulting in loss of mitochondrial connectivity, cristae disorganization, and a vulnerability to ROS-driven injury in the RPE. Concurrently, impaired mitophagy, evidenced by defective PINK1/Parkin signaling and inefficient mitophagosome formation, allows the persistent accumulation of dysfunctional, ROS-producing mitochondria [21,22]. This failure in quality control contributes to the accumulation of intracellular lipofuscin and the extrusion of oxidized lipids and proteins across the basal membrane, forming the core constituents of drusen [8,36]. These convergent abnormalities in mitochondrial morphology, fusion–fission balance, and mitophagy failure are integrated at the subcellular level in Fig. 6, highlighting how mitochondrial fragmentation, cytochrome *c* release, and defective clearance cooperate to drive progressive RPE degeneration. Ultimately, this cascade of bioenergetic crisis, oxidative damage, and inflammatory signaling triggers the demise of the RPE, culminating in the secondary degeneration of photoreceptors and the manifestation of clinical AMD phenotypes, from drusen and pigmentary changes to geographic atrophy. The imbalance between fission, fusion, biogenesis, and mitophagy described above represents the transition from early mitochondrial stress to the chronic degenerative cycle represented in Fig. 6.

## 5 Mitochondrial biogenesis and mitophagy in AMD: A failure of renewal and clearance

The relentless metabolic and oxidative challenges faced by the RPE necessitate a robust system for both generating new mitochondria and eliminating damaged ones. In AMD, this critical cycle of biogenesis and mitophagy is profoundly disrupted, creating a state of energetic deficit and toxic cellular accumulation that propels disease progression [8,24]. As a result, the declining input of newly synthesized mitochondria cannot compensate for the accumulating burden of damaged organelles trapped within undegraded mitophagosomes.

### 5.1 The decline of mitochondrial biogenesis

Mitochondrial biogenesis is the essential counterpoint to degradation, responsible for expanding the mitochondrial network to meet energetic demands. The transcriptional coactivator PGC-1α serves as the master regulator of this process, coordinating the expression of nuclear-encoded mitochondrial genes through transcription factors like NRF-1 and NRF-2 [24]. In the aging RPE and in experimental models of AMD, a significant downregulation of PGC-1α signaling has been consistently observed [22]. The overall consequences of altered PGC-1α–NRF1/NRF2–TFAM signaling for mitochondrial renewal in the RPE are summarized in Fig. 3, which contrasts physiological biogenesis with the pathological impairment observed in AMD. This repression leads to a reduced synthesis of critical ETC components and a decline in the overall mitochondrial mass. The consequence is an inadequate capacity for ATP production, leaving the RPE unable to meet the high energy requirements for phagocytosis, retinoid recycling, and ionic transport. This bioenergetic failure renders the RPE increasingly vulnerable to routine stress and is a direct contributor to the gradual functional decline observed in early AMD [8].

### 5.2 The dual role of mitophagy: compensatory upregulation and late-stage failure

Mitophagy represents a pivotal quality-control checkpoint, and its impairment marks a major turning point in AMD pathogenesis (Fig. 4). Experimental data suggest a biphasic role: an early compensatory upregulation followed by late-stage failure under chronic damage, as detailed below.

#### 5.2.1 Compensatory upregulation in early stress

In the face of initial mitochondrial insult, cells may mount a protective response by upregulating mitophagy to clear damaged organelles. Several studies, often modeling early cellular stress, have observed such an increase in mitophagic activity [37,38]. This surge likely represents an adaptive, protective mechanism aimed at maintaining mitochondrial quality and cellular homeostasis during the initial phases of metabolic or oxidative challenge.

#### 5.2.2 Overwhelmed clearance and late-stage impairment

However, in the chronic setting of AMD, this compensatory mechanism becomes overwhelmed. The persistent high load of damaged organelles may saturate the mitophagy machinery, leading to impaired mitophagic flux, including inefficient Parkin recruitment, mitophagosome maturation, lysosomal degradation, trafficking, and turnover. Furthermore, the overall autophagic–lysosomal pathway itself can become dysfunctional with age, creating a bottleneck in mitophagosome–lysosome fusion and degradation even if initial targeting occurs [38]. Consistent with this, live-cell imaging and mechanistic studies in human RPE cells have shown that AMP-activated protein kinase (AMPK)-dependent regulation of autophagy and mitochondrial dynamics is critical for surviving oxidative stressors such as UVA and sodium iodate, whereas disruption of these pathways (e.g. via PARP-1 overactivation) precipitates mitochondrial disintegration and cell death [[39], [40], [41]]. Consequently, in late-stage AMD and in models using RPE from AMD donors or chronic oxidative stress, a significant impairment in PINK1/Parkin-mediated mitophagy is consistently observed [22,42]. This failure in quality control leads to the persistent accumulation of dysfunctional, ROS-producing mitochondria, creating a vicious cycle of damage.

Therefore, while transient mitophagy induction is beneficial, its sustained activation in the face of an overwhelming burden of damage and a declining lysosomal capacity ultimately proves insufficient. The transition from compensatory mitophagy to its eventual failure is a critical step that propels the accumulation of intracellular waste and drives RPE cell death in advanced AMD.

## 6 Oxidative stress and inflammation: the mitochondrial-inflammatory nexus in AMD

**Fig. 7:**
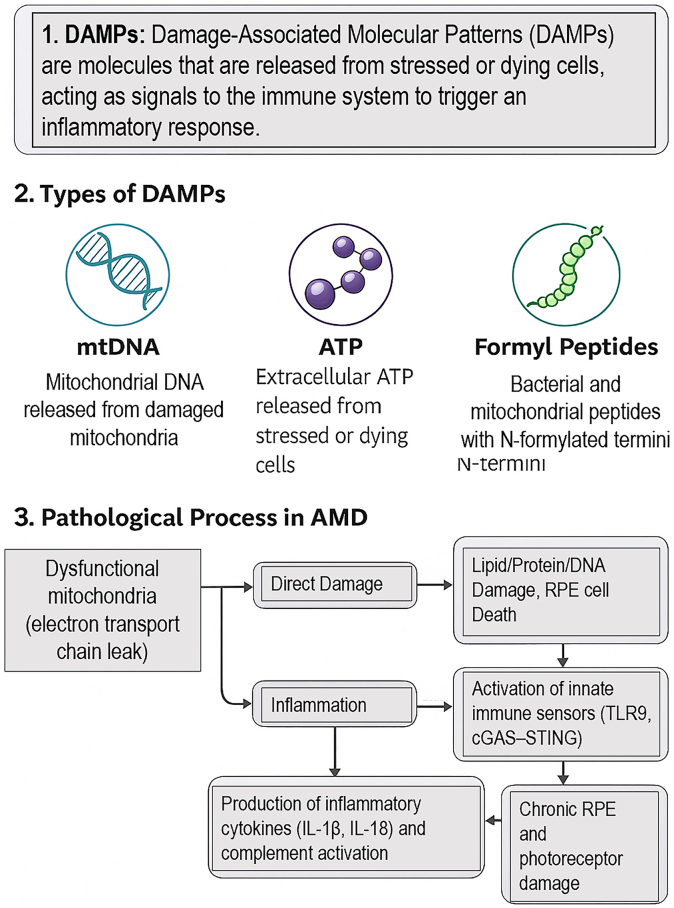
Mitochondrial DAMPs as central mediators linking mitochondrial dysfunction to inflammation and retinal injury in AMD. Mitochondrial DAMPs, including mtDNA, extracellular ATP, and N-formylated peptides, are released from stressed or dying retinal cells as a consequence of electron transport chain dysfunction and oxidative injury. These signals induce direct lipid, protein, and DNA damage in RPE cells and concurrently activate innate immune sensors, particularly TLR9 and the cGAS–STING pathway, leading to sustained production of pro-inflammatory cytokines such as interleukin-1β and interleukin-18 and persistent complement activation. The convergence of direct oxidative injury and chronic inflammation drives progressive RPE and photoreceptor degeneration, reinforcing the pathogenic cycle of tissue damage in AMD.

The bioenergetic and quality control failures within the RPE mitochondrial network create a pro-oxidant intracellular environment that serves as a potent ignition source for the chronic, low-grade inflammation characteristic of AMD. This section delineates the pathway from mitochondrial dysfunction to the sustained inflammatory response that drives disease pathology. The inflammatory mechanisms discussed here follow the sequence depicted in Fig. 7.

### 6.1 From ETC dysfunction to chronic oxidative stress

The primary consequence of a damaged and inefficient mitochondrial population is a leaky ETC. As noted, dysfunctional ETC complexes, particularly Complex I and III, aberrantly transfer electrons to molecular oxygen, resulting in the excessive generation of superoxide anions and other ROS [43,44]. In a healthy RPE, this basal ROS production is counterbalanced by a robust antioxidant system. However, in AMD, the sheer volume of ROS from a failing mitochondrial network overwhelms these defences, leading to a state of chronic oxidative stress. This relentless oxidant burden directly damages cellular constituents, inducing lipid peroxidation, protein carbonylation, and nucleic acid oxidation, which collectively impair vital RPE functions and ultimately trigger apoptotic or necroptotic cell death [8,29]. Several antioxidant interventions have been shown to mitigate this oxidative cascade at the level of the RPE. Polyphenols and carotenoids such as resveratrol, epigallocatechin gallate, lycopene, and lutein attenuate UVA- or growth factor–induced injury in ARPE-19 cells by reducing ROS generation, stabilising mitochondrial function, and suppressing pro-migratory signaling [[45], [46], [47], [48]]. The loss of RPE integrity is a seminal event that disrupts photoreceptor support and initiates the clinical features of AMD.

### 6.2 Mitochondria as instigators of innate immune activation

Beyond causing direct cellular damage, mitochondria play an active and direct role in initiating and amplifying inflammatory cascades. Dysfunctional mitochondria release a suite of damage-associated molecular patterns (DAMPs) that are interpreted by the innate immune system as signals of cellular distress [25]. Key mitochondrial DAMPs (mtDAMPs) include:

• **Cell-free mtDNA:** When released into the cytosol or extracellular space, oxidized mtDNA is a potent ligand for endosomal Toll-like receptor 9 (TLR9) and the cytosolic cGAS–STING pathway. Activation of these pathways triggers the production of type I interferons and pro-inflammatory cytokines [49].
• **N-formyl peptides:** Derived from mitochondrial proteins, these peptides act as chemoattractants for immune cells by binding to formyl peptide receptors.
• **ATP:** When released in large quantities from damaged cells, extracellular ATP acts as a DAMP, signaling through purinergic receptors to incite inflammation.

These mtDAMP-mediated pathways directly correspond to the signaling schema in Fig. 7, where TLR9 and the cGAS–STING axis represent key early sensors of mitochondrial distress. The release of these mtDAMPs from ruptured mitochondria and destabilized mitophagosomes into the cytosol and extracellular space initiates potent pro-inflammatory signaling cascades, creating a direct pathogenic link between organelle failure and tissue-level inflammation. In the context of AMD, the accumulation of mtDAMPs creates a pro-inflammatory microenvironment within the retina and the choroid. This is critically significant given the established genetic link between AMD and polymorphisms in genes of the complement system, such as complement factor H (CFH) [6,50]. The complement system, a key arm of innate immunity, is designed to be activated by "foreign" or "altered-self" surfaces. The inflammatory milieu generated by mtDAMPs can directly engage and overwhelm the regulatory capacity of complement factor H, leading to hyperactivation of the alternative complement pathway on RPE and choroidal cells [26]. This creates a vicious, self-perpetuating cycle: mitochondrial damage induces inflammation and complement activation, which in turn causes further collateral damage to the RPE, resulting in more mitochondrial dysfunction and the release of additional mtDAMPs. This connection is particularly relevant given AMD's genetic architecture. The *CFH* risk variant (Y402H) exhibits reduced binding to host cells, compromising its ability to distinguish "self" from "non-self." In this context, the persistent release of mtDAMPs creates a continuous "danger" signal that overwhelms the already compromised regulatory capacity of CFH, leading to uncontrolled complement activation. This provides a mechanistic link between chronic mitochondrial stress and the complement dysregulation that characterizes AMD pathogenesis in genetically susceptible individuals. Purinergic signaling further amplifies this mitochondrial–inflammatory axis. In mouse models, activation of the ATP-gated ion channel P2X7 contributes to retinal degeneration via coordinated actions in multiple retinal cell types, linking extracellular ATP release, mitochondrial stress, and cell death pathways [40].

This mitochondrial-inflammatory axis provides a unifying pathophysiological framework for AMD. It directly links the initial insults of aging, genetics, and environment to the sustained inflammatory and complement-driven tissue destruction that defines the disease. Consequently, the RPE becomes both the source and the target of an inflammatory attack, with its own dysfunctional organelles acting as the central provocateurs. This transition from innate sensing to complement activation forms the latter half of the sequence illustrated in Fig. 7.

## 7 Clinical and experimental evidence linking mitochondrial dysfunction to AMD

The conceptual framework of mitochondrial failure in AMD is substantiated by a compelling body of evidence derived from *in vitro* models, animal studies, and direct analysis of human tissues. This multi-faceted evidence base firmly establishes aberrant mitochondrial dynamics as a core pathological feature of the disease.

### 7.1 Insights from preclinical models

Cell culture and animal models have been instrumental in delineating the cause-and-effect relationships between specific mitochondrial processes and AMD-like pathology.

• **Oxidative Stress Models:** Exposing human RPE cells to sustained oxidative stress (e.g., with hydrogen peroxide) recapitulates key features of AMD, including increased mitochondrial fission, Drp1 activation, and a concomitant reduction in fusion proteins like Mfn2 [51]. This shift toward a fragmented mitochondrial phenotype is associated with increased apoptosis and the accumulation of lipofuscin-like autofluorescence.
• **Genetic Manipulation of Dynamics:** Experimental knockdown of the fusion protein OPA1 in RPE cells leads to severe mitochondrial fragmentation, ETC dysfunction, and increased susceptibility to oxidative stress-induced cell death [52]. Conversely, genetic or pharmacological inhibition of Drp1 has been shown to attenuate oxidative damage and preserve RPE function in stress models, highlighting the therapeutic potential of modulating fission [53].
• **Mitophagy and Biogenesis Deficits:** Studies using RPE cells from AMD donors or carrying AMD-risk genes have demonstrated impaired PINK1/Parkin-mediated mitophagy and repressed PGC-1α signaling [22,23]. For instance, induced pluripotent stem cell-derived RPE cells from patients with AMD show defective clearance of damaged mitochondria due to impaired mitophagosome maturation and elevated ROS levels, a phenotype that can be partially rescued by enhancing mitophagy or biogenesis [22,52].

### 7.2 Evidence from human studies

Direct analysis of human ocular tissues and biofluids provides the most critical validation of mitochondrial involvement in AMD, with key findings summarized in Table 2.

• **Ultrastructural and Histological Analysis:** Transmission electron microscopy of RPE from human donor eyes with AMD reveals a hallmark finding: significantly enlarged and structurally abnormal mitochondria, often accompanied by the accumulation of lipofuscin and mitochondrial-derived vesicles [58,59]. This swollen morphology, indicative of severe mitochondrial membrane depolarization and osmotic matrix expansion under metabolic stress, may seem paradoxical to the fragmented network observed in experimental models of excessive fission [60,61]. This discrepancy likely reflects different stages or aspects of the pathogenic process; fragmentation may be an early dynamic response to stress, while the swollen, enlarged mitochondria seen in human tissue may represent a later, degenerating state where the organelle is grossly damaged and unable to undergo normal fission/fusion, ultimately leading to the failure of quality control [62,63].
• **Mitochondrial Genomics:** Sequencing of mtDNA from RPE-choroid complexes of AMD donors has identified a higher burden of somatic mtDNA mutations and specific deletions compared to age-matched controls [33]. These mutations, which accumulate with age and are exacerbated by oxidative stress, directly compromise the function of encoded ETC proteins, creating a feed-forward cycle of energetic decline.
• **Biomarker Profiling:** Metabolomic and proteomic studies of serum, aqueous humor, and RPE from AMD patients have revealed distinct signatures of mitochondrial dysfunction. These include elevated levels of circulating cell-free mtDNA, alterations in TCA cycle intermediates, and a downregulation of nuclear-encoded mitochondrial proteins involved in oxidative phosphorylation [50,57].

**Table 2:** Summary of human tissue and biomarker studies supporting the role of mitochondrial dysfunction in AMD.

Type of Evidence	Key Findings in AMD Tissues/Biofluids	Pathophysiological Interpretation	References
**Ultrastructural Analysis**	Enlarged, swollen mitochondria with disrupted cristae; accumulation of lipofuscin.	Indicates mitochondrial stress, fusion attempts, and failure of quality control.	[[54], [55], [56]]
**mtDNA Analysis**	Increased somatic mutations and specific deletions (e.g., "common deletion") in RPE.	Leads to defective ETC complexes, creating a cycle of ROS production and further damage.	[33,44]
**Biomarker Profiling**	Elevated circulating cell-free mtDNA; altered TCA cycle metabolites; downregulation of OXPHOS proteins.	Reflects systemic and local metabolic stress, cell death, and bioenergetic compromise.	[50,57]
**Genetic Association**	AMD-risk genes (e.g., *ARMS2*/*HTRA1*) linked to mitochondrial stress response.	Suggests genetic susceptibility exacerbates mitochondrial vulnerability to environmental insults.	[28,50]

### 7.3 Translational insights from neurodegenerative diseases

The centrality of mitochondrial dysfunction in AMD mirrors its well-established role in other age-related neurodegenerative conditions, such as Alzheimer's and Parkinson's disease. In Parkinson's disease, mutations in PINK1 and Parkin cause early-onset disease due to a failure in mitophagy, leading to the accumulation of damaged mitochondria in dopaminergic neurons [21]. Similarly, in Alzheimer's disease, impaired mitochondrial dynamics and bioenergetics are early features that contribute to synaptic failure and neuronal loss [64]. The parallels are striking: post-mitotic, high-energy cells—whether RPE, neurons, or photoreceptors—are exquisitely vulnerable to defects in mitochondrial quality control. The insights gained from these neurological disorders, including therapeutic strategies aimed at enhancing mitochondrial biogenesis (e.g., with PGC-1α activators) or improving mitophagy, provide valuable roadmaps for developing similar interventions for AMD.

Collectively, this convergence of evidence from bench to bedside solidifies the position of mitochondrial integrity as a critical determinant of RPE health and a compelling target for halting or slowing the progression of AMD.

## 8 Emerging therapeutic strategies targeting mitochondria in AMD

**Fig. 8:**
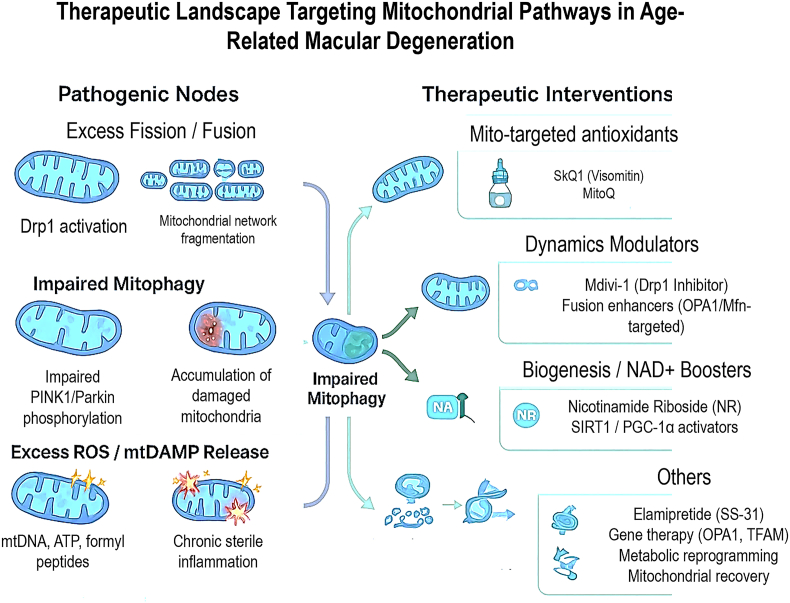
Therapeutic landscape targeting mitochondrial pathways in AMD. The left panel depicts key pathogenic mitochondrial nodes in AMD, including Drp1-driven excessive fission with mitochondrial network fragmentation, impaired PINK1–Parkin–mediated mitophagy with accumulation of damaged mitochondria, and excessive ROS production with release of mitochondrial damage-associated molecular patterns (mtDAMPs) such as mitochondrial DNA, ATP, and N-formyl peptides that sustain chronic sterile inflammation. The right panel summarizes major classes of emerging mitochondrial-targeted interventions, including mitochondria-targeted antioxidants (SkQ1/Visomitin and MitoQ) to neutralize intramitochondrial ROS, mitochondrial dynamics modulators (Mdivi-1 as a Drp1 inhibitor and OPA1/mitofusin-targeted fusion enhancers) to restore network integrity, biogenesis and NAD+ boosters (nicotinamide riboside and SIRT1/PGC-1α activators) to replenish mitochondrial mass and function, and additional strategies such as elamipretide (SS-31), gene therapies targeting OPA1 and TFAM, and metabolic reprogramming approaches aimed at promoting mitochondrial recovery and retinal cell survival.

The unequivocal role of mitochondrial dysfunction in AMD pathogenesis has catalysed a paradigm shift in therapeutic development, moving beyond managing late-stage complications to targeting fundamental cellular mechanisms of the disease. A new generation of interventions aimed at bolstering mitochondrial health is now under rigorous investigation, offering potential strategies to slow or halt the progression of AMD, particularly the currently untreatable dry form (Fig. 8).

### 8.1 Pharmacological agents targeting mitochondrial pathways

Current research is focused on several pharmacological classes designed to intervene at specific points of mitochondrial failure:

• **Advanced Antioxidants:** Moving beyond broad-spectrum supplements like the AREDS2 formula, next-generation compounds are designed to selectively accumulate within mitochondria to neutralize ROS at its primary source. Molecules such as MitoQ and SKQ1 (Visomitin) are conjugated to a lipophilic cation (triphenylphosphonium), enabling them to cross lipid bilayers and reach high concentrations in the mitochondrial matrix. Early-phase clinical trials have suggested that topical SKQ1 may improve parameters of retinal health, and recent 2024 preclinical studies show it suppresses AMD-like retinopathy progression in animal models by inhibiting p38MAPK and ERK1/2 signaling pathways, mechanisms central to mitochondrial stress responses [65,66]. However, the translational path for many mitochondrial antioxidants remains challenging.
• **Modulators of Mitochondrial Dynamics:** Correcting the imbalance between fission and fusion represents a promising strategy. Small-molecule inhibitors of Drp1, such as Mdivi-1, have demonstrated efficacy in preclinical models by reducing excessive mitochondrial fragmentation, attenuating oxidative stress, and promoting RPE cell survival [53]. Conversely, compounds that enhance the activity of fusion proteins like OPA1 or mitofusins are also being explored to restore mitochondrial networking and bioenergetic efficiency [52].
• **Enhancers of Mitophagy and Biogenesis:** Strategies to clear damaged organelles and stimulate the generation of healthy ones are at the forefront of therapeutic discovery. Compounds that activate the PINK1-Parkin pathway or bypass its deficits to induce mitophagy are in early development. Nicotinamide riboside (NR), a NAD+ precursor that enhances PGC-1α-mediated mitochondrial biogenesis, has demonstrated robust protective effects in preclinical models of retinal degeneration. Systemic NR treatment prevents light-induced photoreceptor loss by maintaining retinal NAD+ levels and reducing oxidative stress-induced apoptosis, with several clinical trials now underway to validate these findings in AMD patients [67].

A consolidated overview of the major mitochondrial-targeted therapeutic agents, their mechanistic targets, and current stage of development is summarized in Table 3.

**Table 3:** Emerging therapies targeting mitochondrial dysfunction in AMD.

Agent/Strategy	Primary Target/Mechanism	Level of Evidence	Disease Context/Model	Key Outcomes	Clinical Status/Trial ID	References
SkQ1 (Visomitin)	Mito-targeted antioxidant	In vitro, in vivo, early clinical	Ocular surface disease; preclinical AMD-related oxidative stress models	↓ROS, improved RPE survival	Clinical development in dry eye disease; preclinical evidence in AMD-related models	[48,49]
MitoQ	Mito-targeted ubiquinone antioxidant	In vitro, in vivo	Oxidative stress RPE	↓mtROS, preserved Δψm	No AMD trials	[23]
Elamipretide	Inner membrane stabilizer	Phase II	Non-exudative AMD/GA	Improved mitochondrial function	ReCLAIM-2	[48]
Nicotinamide riboside	NAD+ booster, biogenesis enhancer	In vitro, in vivo	Light-induced degeneration	↑NAD+, ↓apoptosis	Early clinical	[50]
Mdivi-1 (Drp1 inhibitor)	Fission inhibition	In vitro, in vivo	RPE oxidative stress	↓fragmentation, ↑viability	Preclinical	[43]
OPA1/Mfn2 activation	Fusion enhancement	Preclinical	Fusion-deficient RPE	Restored ATP, ↓fragmentation	Preclinical	[18,42]
PINK1/Parkin activators	Mitophagy restoration	Preclinical	AMD iPSC-RPE	Improved mitophagic flux	Preclinical	[21,22,37]
PGC-1α activators	Biogenesis induction	Preclinical	Aged/AMD RPE	↑OXPHOS, ↑biogenesis	Preclinical	[22,24]
Gene therapy (OPA1, TFAM)	Mitochondrial resilience	Preclinical	RPE gene delivery	↓oxidative injury	Translational	[51]

ΔΨm, mitochondrial membrane potential

### 8.2 Gene therapy and novel biological approaches

For monogenic forms of retinal degeneration and potentially for AMD, gene therapy offers a transformative, one-time intervention.

• **Gene Therapy:** Approaches under consideration include delivering genes that encode for key mitochondrial quality control proteins, such as *OPA1* or *TFAM*, directly to the RPE to enhance its resilience. While no clinical trials for AMD-specific mitochondrial gene therapies are yet active, the proven success of RPE65 gene therapy for Leber congenital amaurosis provides a robust technical and regulatory foundation for such endeavors [68].
• **Metabolic Reprogramming and Neuroprotection:** Interventions aimed at improving overall cellular metabolism are also being pursued. Systemic administration of elamipretide was investigated in the Phase II ReCLAIM-2 trial for non-exudative AMD with geographic atrophy (NCT03891875). In the published Phase II report, elamipretide did not meet the prespecified primary efficacy endpoints; however, exploratory analyses provided signals consistent with biologic activity in selected structural and visual function measures, including ellipsoid zone related outcomes, underscoring the challenges of translating mitochondrial-targeted therapeutics into clinically meaningful benefit in AMD [65].

### 8.3 Unresolved questions

Despite this promising outlook, significant challenges remain. A primary hurdle is the precise and targeted delivery of therapeutics to the RPE and photoreceptors without causing systemic side effects. The development of novel delivery vehicles, including cell-penetrating peptides and refined viral vectors, is critical. Furthermore, the field must address the inherent complexity of mitochondrial dynamics; therapeutic intervention must be finely tuned, as excessive fusion or unchecked mitophagy could be as detrimental as the deficits they aim to correct.

Key unresolved questions guide future research: What is the optimal timing for intervention in the decades-long course of AMD? Can a single agent effectively target the intertwined pathways of dynamics, biogenesis, and mitophagy, or will combination therapies be necessary? Ongoing and future clinical trials, coupled with biomarker development to identify patients with a predominant "mitochondriopathic" phenotype, will be essential to translate these compelling preclinical strategies into effective, personalized treatments for AMD.

### 8.4 Limitations of this review and translational challenges

While this review synthesizes the compelling case for mitochondrial dysfunction in AMD, several limitations and translational challenges must be acknowledged. Firstly, the evidence base is predominantly derived from preclinical models, which may not fully recapitulate the chronic, multifactorial nature of human AMD. Secondly, significant heterogeneity exists among AMD subtypes, and it remains unclear whether mitochondrial failure is a universal driver or specific to certain patient endophenotypes.

From a therapeutic perspective, translating these strategies faces considerable hurdles. These include the poor bioavailability and targeted delivery of compounds to the RPE and photoreceptors, the potential for off-target systemic effects, and the delicate balance required when modulating dynamic processes—where excessive fusion or mitophagy could be as detrimental as their deficiency.

Finally, this review has focused on the mitochondrial axis, but a complete understanding of AMD pathogenesis requires the integration of this pathway with other key systems, including lipid metabolism, the autophagy-lysosomal network, and the complement system. Future research must strive to build unified models that account for the complex interplay between these pathways to identify the most effective nodal points for therapeutic intervention.

## 9 Future perspectives

The trajectory of research into mitochondrial dynamics in AMD points toward an era of increasingly precise and mechanism-driven therapeutics. Several key avenues will shape future investigations. First, the development of sensitive and specific biomarkers for in vivo assessment of mitochondrial health is paramount for advancing precision medicine in AMD. Beyond conventional optical coherence tomography (OCT), adaptive optics (AO) retinal imaging employs real-time wavefront correction to compensate for higher-order ocular aberrations, thereby enabling near-cellular resolution visualization of retinal microstructures. When integrated with OCT or scanning laser ophthalmoscopy (AO-OCT, AO-SLO), this technology permits detailed interrogation of photoreceptor inner segments, which are densely populated by mitochondria and critically dependent on intact mitochondrial dynamics and bioenergetics. AO-based imaging can detect alterations in retinal reflectance and microstructural patterning, enabling high-resolution phenotyping in intermediate and late dry AMD. Mechanistically, a mitochondrial contribution to outer retinal reflectivity is supported by OCT-histology correlation literature, but the attribution of specific AO reflectivity signatures to discrete mitochondrial structural states should be framed as a biologically informed hypothesis unless directly validated in AMD cohorts with matched molecular or ultrastructural endpoints. Accordingly, AO-derived reflectivity metrics may serve as a pragmatic, non-invasive surrogate endpoint for outer retinal integrity and may support early detection, patient stratification, and longitudinal monitoring in trials of mitochondrial-targeted interventions, while requiring continued validation for mitochondrial specificity [69,70]. Such stratification would facilitate personalized medicine approaches, allowing therapies to be matched to an individual's specific “mitochondriopathic” profile. Given the emergence of this potential AMD endophenotype, candidate biomarkers of mitochondrial dysfunction are summarized in Table 4, providing a foundation for future patient stratification and personalized interventions.

**Table 4:** Candidate biomarkers reflecting mitochondrial dysfunction in age-related macular degeneration (AMD).

Biomarker	Sample Type	Process Reflected	AMD Findings	Clinical Utility	References
cf-mtDNA	Serum, plasma, aqueous	mtDNA integrity	↑cf-mtDNA	Stratification, response monitoring	[40,46]
mtDNA deletions	RPE–Bruch's tissue	mtDNA stability	↑somatic deletions	Stage marker, GA correlation	[31,38]
OXPHOS proteins	RPE proteomics	ETC function	↓complex I/III/IV	Identify ETC-deficient patients	[40,45]
TCA metabolites	Serum metabolomics	Metabolic flux	Altered succinate/fumarate	Systemic biomarker	[46]
Lipofuscin/A2E	RPE imaging	Mitophagy/autophagy	↑lipofuscin	Early AMD marker	[8,34]
Ellipsoid zone integrity	OCT/AO imaging	Photoreceptor mitochondrial health	EZ disruption	Imaging surrogate endpoint	[15]
AO-reflectivity markers	Adaptive optics	Mitochondrial structure	Altered reflectance	High-resolution phenotyping	[62]
Oxidative markers (8-OHdG, MDA)	Aqueous humor	Oxidative stress	Elevated oxidative damage	Adjunct oxidative biomarker	[29]

Second, the intricate crosstalk between mitochondrial dynamics and other cellular pathways, particularly inflammasome activation and the integrated stress response, requires deeper elucidation. Understanding these networks will reveal novel nodal points for therapeutic intervention. Furthermore, the potential of combination therapies—for instance, pairing a mitophagy enhancer with a mitochondrial antioxidant—should be systematically explored to address the multifactorial nature of mitochondrial decline. Finally, the repurposing of mitochondrial therapies from other neurodegenerative conditions, validated by robust preclinical models that accurately recapitulate the chronicity of AMD, represents a promising and efficient strategy to accelerate the drug development pipeline.

## 10 Conclusion

The accumulation of evidence from cellular, animal, and human studies solidifies the position of mitochondrial dysfunction as a cornerstone of AMD pathogenesis. The RPE, with its exceptionally high metabolic demands, is exquisitely vulnerable to age- and stress-mediated declines in mitochondrial quality control. The core processes of fission, fusion, biogenesis, and mitophagy, which maintain a healthy and functional mitochondrial network, become profoundly dysregulated in AMD. This failure leads to a cascade of bioenergetic deficit, excessive oxidative stress, and the release of damage-associated molecular patterns that fuel a chronic inflammatory and complement-mediated attack on the retina.

This refined understanding shifts the view of AMD from a purely degenerative or vascular disorder to one that is fundamentally rooted in cellular energetics and quality control. While anti-VEGF therapies remain a mainstay for neovascular AMD, the pressing unmet need in geographic atrophy has directed the field's focus toward these upstream pathological mechanisms. The emergence of mitochondrial-targeted strategies—including stabilizers of dynamics, enhancers of turnover, and advanced gene therapies—heralds a transformative potential for clinical management. By targeting the root causes of RPE and photoreceptor demise, these innovative approaches offer the promise of preserving vision and altering the progressive course of this blinding disease.

## Ethics declarations

### Ethics approval and consent to participate

This study does not involve any new human participants, human data, or human tissue. Therefore, no Institutional Review Board (IRB) or Ethics Committee approval was required, and the Human Ethics and Consent to Participate declarations are not applicable.

## Consent for publication

Not applicable. This article does not contain any individual person's data in any form.

## Availability of data and materials

Not applicable. No new datasets were generated or analysed for this review.

## Declaration on integrity and transparency

The authors confirm that no paper mill and no artificial intelligence was used in the generation or submission of this manuscript.

## Précis

This review synthesizes evidence that disrupted mitochondrial fission, fusion, biogenesis, and mitophagy drive retinal pigment epithelium failure, chronic inflammation, and vision loss in AMD, highlighting mechanism-based therapies and biomarker-guided precision care.

## Funding

No specific funding was received from any funding bodies in the public, commercial, or not-for-profit sectors to conduct the work described in this manuscript.

## CRediT authorship contribution statement

**Kai-Yang Chen:** Conceptualization, Investigation, Methodology, Software, Supervision, Validation, Visualization, Writing – original draft, Writing – review & editing. **Hoi-Chun Chan:** Conceptualization, Investigation, Methodology, Supervision, Writing – original draft, Writing – review & editing. **Wan-Wan Lin:** Writing – review & editing. **Chi-Ming Chan:** Conceptualization, Investigation, Methodology, Project administration, Software, Supervision, Validation, Visualization, Writing – original draft, Writing – review & editing.

## Declaration of competing interest

The authors declare no conflicts of interest related to this study. The research was conducted independently, without any financial or personal relationships that could influence the work reported in this manuscript. No funding from pharmaceutical companies or other entities with vested interests was received. All authors affirm that the findings and conclusions are solely their own and are not influenced by external interests.

## Data Availability

Data availability: Not applicable. No new datasets were generated or analysed for this review.

## List of abbreviations

**AMD**: age-related macular degeneration
**AMPK**: AMP-activated protein kinase
**AREDS2**: Age-Related Eye Disease Study 2
**ARMS2**: age-related maculopathy susceptibility 2
**ARMS2/HTRA1**: age-related maculopathy susceptibility 2 / high-temperature requirement A serine peptidase 1 locus
**ARPE-19**: human retinal pigment epithelial cell line ARPE-19
**ATP**: adenosine triphosphate
**C1q**: complement component 1q
**C3**: complement component 3
**C3a**: complement component 3a
**C3b**: complement component 3b
**C5a**: complement component 5a
**C5b-9**: complement terminal complement complex C5b-9 (membrane attack complex)
**CFH**: complement factor H
**cGAS**: cyclic GMP-AMP synthase
**cGAS-STING**: cyclic GMP-AMP synthase–stimulator of interferon genes signaling axis
**DAMPs**: damage-associated molecular patterns
**DNA**: deoxyribonucleic acid
**Drp1**: dynamin-related protein 1
**ETC**: electron transport chain
**GA**: geographic atrophy
**GTP**: guanosine triphosphate
**HTRA1**: high-temperature requirement A serine peptidase 1
**IL-1β**: interleukin-1 beta
**IL-18**: interleukin-18
**IMM**: inner mitochondrial membrane
**LC3**: microtubule-associated protein 1 light chain 3
**MAC**: membrane attack complex
**MASP-2**: mannose-binding lectin-associated serine protease 2
**MBL**: mannose-binding lectin
**MerTK**: Mer tyrosine kinase receptor
**Mdivi-1**: mitochondrial division inhibitor 1 (small-molecule Drp1 inhibitor)
**MFF/Mff**: mitochondrial fission factor
**Mfn1**: mitofusin 1
**Mfn1/2**: mitofusin 1/2 (shorthand referring to both mitofusin 1 and mitofusin 2)
**Mfn2**: mitofusin 2
**MiD49/51**: mitochondrial dynamics proteins of 49 and 51 kDa
**MitoQ**: mitoquinone (mitochondria-targeted ubiquinone antioxidant)
**mtDAMPs**: mitochondrial damage-associated molecular patterns
**mtDNA**: mitochondrial DNA
**NAD+**: nicotinamide adenine dinucleotide (oxidized form)
**NCT03891875**: ClinicalTrials.gov identifier NCT03891875 (ReCLAIM trial)
**NLRP3**: NOD-like receptor family pyrin domain-containing 3
**NR**: nicotinamide riboside
**NRF1**: nuclear respiratory factor 1
**NRF2**: nuclear respiratory factor 2
**NRF1/NRF2**: nuclear respiratory factor 1/2
**OPA1**: optic atrophy 1
**OMM**: outer mitochondrial membrane
**OXPHOS**: oxidative phosphorylation
**P2X7**: P2X7 purinergic receptor
**Parkin**: E3 ubiquitin-protein ligase Parkin
**PARP-1**: poly(ADP-ribose) polymerase-1
**PGC-1α**: peroxisome proliferator-activated receptor-gamma coactivator-1 alpha
**PET**: positron emission tomography
**PINK1**: PTEN-induced kinase 1
**ReCLAIM-2**: phase II randomized clinical trial of elamipretide in non-exudative age-related macular degeneration with geographic atrophy (ClinicalTrials.gov: NCT03891875)
**RPE**: retinal pigment epithelium
**RPE65**: etinal pigment epithelium-specific 65 kDa protein
**ROS**: reactive oxygen species
**SIRT1**: sirtuin 1
**SkQ1**: SkQ1 (plastoquinone-derived mitochondria-targeted antioxidant, Visomitin)
**SS-31**: SzetoSchiller peptide 31 (elamipretide)
**STING**: stimulator of interferon genes
**TCA**: tricarboxylic acid (cycle)
**TFAM**: mitochondrial transcription factor A
**TLR9**: Toll-like receptor 9
**UVA**: ultraviolet A
**VEGF**: vascular endothelial growth factor
**Y402H**: tyrosine-to-histidine substitution at position 402 in complement factor H (CFH Y402H variant)
**ΔΨm**: Mitochondrial membrane potential
